# First-principles calculations of magnetic properties for analysis of magnetization processes in rare-earth permanent magnets

**DOI:** 10.1080/14686996.2021.1947119

**Published:** 2021-09-07

**Authors:** Hiroki Tsuchiura, Takuya Yoshioka, Pavel Novák, Johann Fischbacher, Alexander Kovacs, Thomas Schrefl

**Affiliations:** a Department of Applied Physics, Tohoku University, Sendai, Japan; b Center for Spintronics Research Network (CSRN), Tohoku University, Sendai, Japan; c Institute of Physics, Academy of Sciences of the Czech Republic, Prague, Czech Republic; d Department for Integrated Sensor Systems, Danube University Krems, Krems, Austria

**Keywords:** Rare-earth permanent magnets, crystal field theory, first-principles calculations, atomistic spin model, micromagnetic simulations, 40 Optical, magnetic and electronic device materials, 106 Metallic materials; 401 1st principles methods, 404 Dynamics simulations, 406 Multi-scale / multi-physics modelling

## Abstract

It has been empirically known that the coercivity of rare-earth permanent magnets depends on the size and shape of fine particles of the main phase in the system. Also, recent experimental observations have suggested that the atomic-scale structures around the grain-boundaries of the fine particles play a crucial role to determine their switching fields. In this article, we review a theoretical attempt to describe the finite temperature magnetic properties and to evaluate the reduction of the switching fields of fine particles of several rare-earth permanent magnetic materials based on an atomistic spin model that is constructed using first-principles calculations. It is shown that, over a wide temperature range, the spin model gives a good description of the magnetization curves of rare-earth intermetallic compounds such as *R*_2_Fe_14_B (*R*= Dy, Ho, Pr, Nd, Sm) and SmFe_12_. The atomistic spin model approach is also used to describe the local magnetic anisotropy around the surfaces of the fine particles, and predicts that the rare-earth ions may exhibit planar magnetic anisotropy when they are on the crystalline-structure surfaces of the particles. The dynamical simulation of the atomistic spin model and the corresponding micromagnetic simulation show that the planar surface magnetic anisotropy causes a reduction in the switching field of fine particles by approximately 20–30%, which may be relevant to the atomic-scale surface effects found in the experimental studies.

## Graphical abstract

**Figure:**
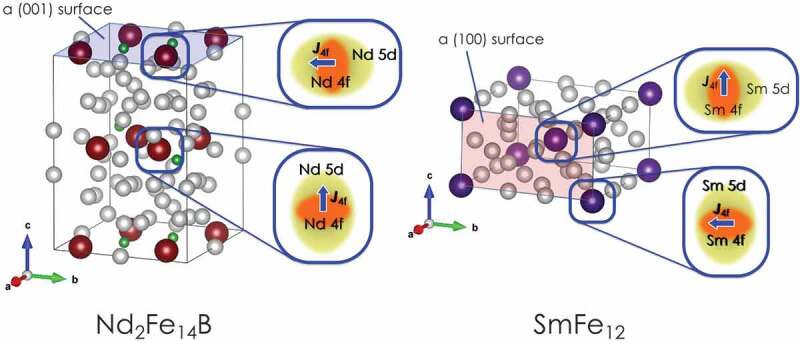

## 1. Introduction

Since the discovery of SmCo${\;}_{5}$-based magnets, the magnetic properties of rare-earth intermetallic compounds have garnered practical interest and become a subject of theoretical research in fundamental physics. To date, several other permanent magnetic materials have been employed in practical applications, such as Nd_2_Fe_14_B, Sm_2_Co_17_, and Sm${\;}_{2}$Fe${\;}_{17}$N${\;}_{3}$. Furthermore, SmFe${\;}_{12}$ has recently attracted significant attention as a promising candidate for new permanent magnetic materials. Many studies on these rare-earth-based materials have been conducted, and the progress in experimental studies is particularly remarkable. However, the theoretical understanding of coercivity in these materials is insufficient to quantitatively describe the coercive force of existing permanent magnets or to predict the magnetization reversal processes in new permanent magnets. This is owing to the difficulties in the theoretical analysis of the hysteresis phenomena, which involve multi-scale physical mechanisms.

At this stage, an important theoretical issue that needs to be addressed is the construction of a model that can accurately describe the equilibrium magnetic properties of rare-earth-based magnetic materials. First-principles calculation techniques provide the best descriptions of the electron states of materials. However, this approach only yields results at a temperature of absolute zero. Moreover, even modern first-principles calculation techniques are limited when considering 4*f* electrons, and some additional corrections, such as the LDA+U method or the self-interaction correction (SIC), are required. Nevertheless, a convenient and reasonably reliable method exists for materials that contain well-localized 4*f* electrons. This method is to estimate magnetocrystalline anisotropy via the crystal field theory, based on first-principles calculations using the so-called open-core method [1]. An effective spin model can then be constructed, which includes the crystal field Hamiltonian for rare-earth ions to describe the finite temperature magnetic properties of rare-earth-based magnetic materials [2]. Intuitively, this method performs better for materials with heavier rare-earth elements, because their 4*f* electrons are more localized, known as lanthanide contraction. We recently identified that this method provides a quantitatively satisfactory description of finite temperature magnetic properties, especially for heavier rare-earth compounds, such as Ho${\;}_{2}$Fe${\;}_{14}$B and Dy${\;}_{2}$Fe${\;}_{14}$ [3]. However, it yields somewhat limited descriptions for lighter rare-earth elements, where 4*f* localization is not sufficiently guaranteed. We review the present status of these model calculations later.

Subsequent to the development of an effective spin model, the next task that needs to be addressed is establishing a description of the magnetization reversal processes. Recently, a series of experimental studies on Nd-Fe-B sintered magnets [4] and Sm(Fe${\;}_{1-x}$Co${\;}_{x}$)${\;}_{12}$ thin films [5] revealed that the atomic-scale structure and elemental distribution around the grain boundaries in these materials have a significant influence on their coercivity. This implies that the local electron state around the surfaces of fine particles is an important factor that affects the switching fields of these materials. Thus, a detailed study of the electron states around the surfaces is expected to improve the theoretical understanding of the switching field, and thus, the coercivity. We believe that the effective spin model is suitable for describing local magnetic properties around surfaces at the atomic scale.

Motivated by the abovementioned experimental observations, several studies have investigated local electron states of the (001) and (100) surfaces of crystalline Nd_2_Fe_14_B based on first-principles calculations. The results showed that Nd ions located on the (001) surface of Nd_2_Fe_14_B not only lose their uniaxial magnetic anisotropy but also exhibit strong planar anisotropy [6–8]. Atomistic spin dynamics simulations were also used to identify that the switching field of a Nd_2_Fe_14_B fine particle is reduced by 70% of the anisotropy field *H*_A_, owing to the local planar magnetic anisotropy around the (001) surface [2,9].

In this study, we first briefly review the current status of methods used to develop the effective spin model of rare-earth intermetallic compounds based on first-principles calculations. Subsequently, we review the surface magnetic anisotropy of crystalline Nd_2_Fe_14_B, and we investigate the same problem for SmFe_12_. We show that the local magnetic anisotropy around the surfaces of these two systems differs significantly. We then consider the switching fields of the fine particles of both systems using the atomistic spin dynamics simulation method. Finally, we discuss the dependence of the switching fields on the particle size based on the micromagnetic simulation technique [10,11], where the parameters are derived by applying a coarse grain to the atomistic spin model.

## 2. A model Hamiltonian

The standard model for describing the contribution of the 4*f* orbital to the magnetocrystalline anisotropy energy is a rare-earth single-ion Hamiltonian [1], and that for the *i*-th ion in the crystal is given as [12]

(1) $${\hat{H}}_{eff,i}=\lambda {\hat{S}}_{i}\cdot {\hat{L}}_{i}+2{\hat{S}}_{i}\cdot H_{m,i}(T)+\;\sum_{l,m}\frac{A_{l,i}^{m}\langle r^{l}\rangle }{a_{l,m}}\sum_{j=1}^{n_{4f}}t_{l}^{m}({\hat{\theta }}_{j},{\hat{\phi }}_{j}),$$

where ${\hat{L}}_{i}(S_{i})$ is the orbital (spin) moment, $H_{m,i}$ is the molecular field at a finite temperature T, $A_{l,i}^{m}\langle r^{l}\rangle$ is the crystal field (CF) parameter, $t_{l}^{m}({\hat{\theta }}_{j},{\hat{\phi }}_{j})$ is the tesseral harmonic function, ${\hat{\theta }}_{j}$ and ${\hat{\phi }}_{j}$ are angle operators for the *j*-th 4*f* electron, and $a_{l,m}$ is the numerical factor [13]. The summation for *j* is taken over the 4*f* electrons, the total number of which is denoted by $n_{4f}$. If we project this Hamiltonian onto the lowest J subspace, the truncated Hamiltonian is expressed as

(2) $${\hat{H}}_{eff,i}^{J}=2(g_{J}-1)H_{m,i}(T)\cdot {\hat{J}}_{i}+\;\sum_{l,m}A_{l,i}^{m}\langle r^{l}\rangle \Theta_{l}{\hat{O}}_{l,i}^{m},$$

where ${\hat{J}}_{i}$ is the total angular momentum, $g_{J}$ is the Lande g-factor, and $\Theta_{l}$ and ${\hat{O}}_{l,i}^{m}$ are the Stevens factor and operators, respectively [14]. The CF Hamiltonian for the 4f electrons is the last term of Equations (1) and (2).

According to previous studies by Novák and Diviš [15–17], the CF coefficients $A_{l}^{m}\langle r^{l}\rangle$ are given by

(3) $$A_{l}^{m}\langle r^{l}\rangle =a_{lm}\int_{0}^{R_{MT}}drr^{2}|R_{4f}(r){|}^{2}V_{l}^{m}(r),$$

where $V_{l}^{m}(r)$ is the component of the total Coulomb potential of a rare-earth ion within an atomic sphere of radius $R_{MT}$. $R_{4f}(r)$ describes the radial shape of the localized 4*f* charge density of rare-earth ions.

In rare-earth permanent magnetic materials, the second term of (2) is much smaller than the first term. Thus, one may assume that the magnetic moments of the rare-earth and 3d transition metal ions are parallel. Under this assumption, we can equate the local anisotropy energy of the i-th R ion with the single ion-free energy defined by the Hamiltonian (2) given as

(4) $$F_{A,i}^{R}(\theta ,\phi ,T)=-k_{B}T\ln Tr\;exp\left[-\frac{{\hat{H}}_{eff,i}^{\left(J\right)}(\theta ,\phi ,T)}{k_{B}T}\right].$$

Using the above $F_{A}^{R}(\theta ,\phi ,T)$, the free energy of the effective spin model is expressed as [2]

(5) $$\begin{array}{rl} & F(\theta ,\phi ,T)=\sum_{i}F_{A,i}^{R}(m_{i}^{R})+\sum_{i}F_{A,i}^{Fe}(m_{i}^{Fe}),\\ & \;\;\;\;\;\;\;\;\;\;\;\;\;\;\;\;\;\;\;\;\;-J_{FeFe}\sum_{i,j}m_{i}^{Fe}\cdot m_{j}^{Fe}-J_{RFe}\sum_{i,j}m_{i}^{R}\cdot m_{j}^{Fe}\\ & \;\;\;\;\;\;\;\;\;\;\;\;\;\;\;\;\;\;\;\;\;-\left[\sum_{i}m_{i}^{R}+\sum_{i}m_{i}^{Fe}\right]\cdot H_{ext}, \end{array}$$

where $m_{i}^{R(Fe)}$ is the magnetic moment of the i-th R (Fe) ion at finite temperatures, $J_{XY}$ is the exchange coupling constant between moments on the X and Y ions, and $H_{ext}$ is the external magnetic field. The atomistic Landau-Lifshitz-Gilbert (LLG)-type equation is obtained as

(6) $$\begin{array}{rl} & \frac{dm_{i}^{X}(T)}{dt}=-\gamma_{i}m_{i}^{X}(T)\times H_{i}^{eff}(T)+\frac{m_{i}^{X}(T)}{m_{i}^{X}(T)}\\ & \;\;\;\;\;\;\;\;\;\;\;\;\;\;\;\;\;\;\;\;\;\;\;\;\times \left(m_{i}^{X}(T)\times \frac{dm_{i}^{X}(T)}{dt}\right), \end{array}$$

with

(7) $$H_{i}^{eff}(T)=-\nabla_{m_{i}}F(T),$$

where X represents R or Fe, and $\gamma_{i}$ is the gyromagnetic ratio of the i-th ion.

Using symmetry considerations, $F_{A}^{R}(\theta ,\phi ,T)$ for the Sm ions on the (100) surface can be expanded as follows:

(8) $$F_{A}^{R}(\theta ,\phi ,T)={\tilde{K}}_{1}(\phi ,T)\sin^{2}\theta +{\tilde{K}}_{2}(\phi ,T)\sin^{4}\theta +{\tilde{K}}_{3}(\phi ,T)\sin^{6}\theta +\cdots ,$$

(9) $${\tilde{K}}_{1}(\phi ,T)=[K_{1}(T)+K{{\;}^{\prime }}_{1}(T)\cos2\phi ],$$

(10) $${\tilde{K}}_{2}(\phi ,T)=[K_{2}(T)+K{{\;}^{\prime }}_{2}(T)\cos2\phi +K{\;}^{\prime }{{\;}^{\prime }}_{2}(T)\cos4\phi ],$$

(11) $${\tilde{K}}_{3}(\phi ,T)=[K_{3}(T)+K{{\;}^{\prime }}_{3}(T)\cos2\phi +K{\;}^{\prime }{{\;}^{\prime }}_{3}(T)\cos4\phi +K{{\;}^{\prime \prime \prime }}_{3}(T)\cos6\phi ].$$

Practically, the coefficients $K_{i}^{\left({\;}^{\prime },{\;}^{\prime }{\;}^{\prime }\right)}(T)$ can be estimated via a comparison between the Taylor series expansion of Equations (4) and (8) [18]. We confirmed that the third and subsequent terms on the right-hand side of Equation (8) are negligibly small for higher temperatures, i.e. T>300 K. Thus, in the LLG simulation, we consider up to the second term of Equation (8).

By contrast, using Kuz’min’s linear theory [19], we obtain analytical expressions for anisotropy constants $K_{i}^{\left({\;}^{\prime },{\;}^{\prime }{\;}^{\prime }\right)}(T)$ as follows:

(12) $$K_{1}(T)=-3A_{2}^{0}\langle r^{4}\rangle \Theta_{2}J^{2}B_{J}^{2}(x)-40A_{4}^{0}\langle r^{4}\rangle \Theta_{4}J^{4}B_{J}^{4}(x)-168A_{6}^{0}\langle r^{6}\rangle \Theta_{6}J^{6}B_{J}^{6}(x),$$

(13) $$K{{\;}^{\prime }}_{1}(T)=A_{2}^{2}\langle r^{2}\rangle \Theta_{2}J^{2}B_{J}^{2}(x)+6A_{4}^{2}\langle r^{4}\rangle \Theta_{4}J^{4}B_{J}^{4}(x)+15A_{6}^{2}\langle r^{6}\rangle \Theta_{6}J^{6}B_{J}^{6}(x),$$

(14) $$K_{2}(T)=35A_{4}^{0}\langle r^{4}\rangle \Theta_{4}J^{4}B_{J}^{4}(x),+378A_{6}^{0}\langle r^{6}\rangle \Theta_{6}J^{6}B_{J}^{6}(x),$$

(15) $$K{{\;}^{\prime }}_{2}(T)=-7A_{4}^{2}\langle r^{4}\rangle \Theta_{4}J^{4}B_{J}^{4}(x)-48A_{6}^{2}\langle r^{6}\rangle \Theta_{6}J^{6}B_{J}^{6}(x),$$

(16) $$K{{\;}^{\prime \prime }}_{2}(T)=A_{4}^{4}\langle r^{4}\rangle \Theta_{4}J^{4}B_{J}^{4}(x)+10A_{6}^{4}\langle r^{6}\rangle \Theta_{6}J^{6}B_{J}^{6}(x),$$

where $J^{l}B_{J}^{l}(x)$ is the generalized Brillouin function [19] with $x=2J|g_{J}-1|H_{m}/k_{B}T$.

## 3. Bulk magnetic properties

We first investigate the bulk magnetic properties under equilibrium, obtained via effective spin models, for several rare-earth intermetallic compounds. The emphasis in this study is on a physical discussion; mathematical and numerical details are available in existing literature [2,3,8,20,21].

### $R_{2}$Fe${\;}_{14}$B systems

3.1.

Because $R_{2}$Fe${\;}_{14}$B systems can be synthesized for almost all rare-earth elements, we compare their magnetic properties with respect to the differences between rare-earth elements. This is a convenient approach to confirm the validity of the effective spin model method.

**Figure 1.**
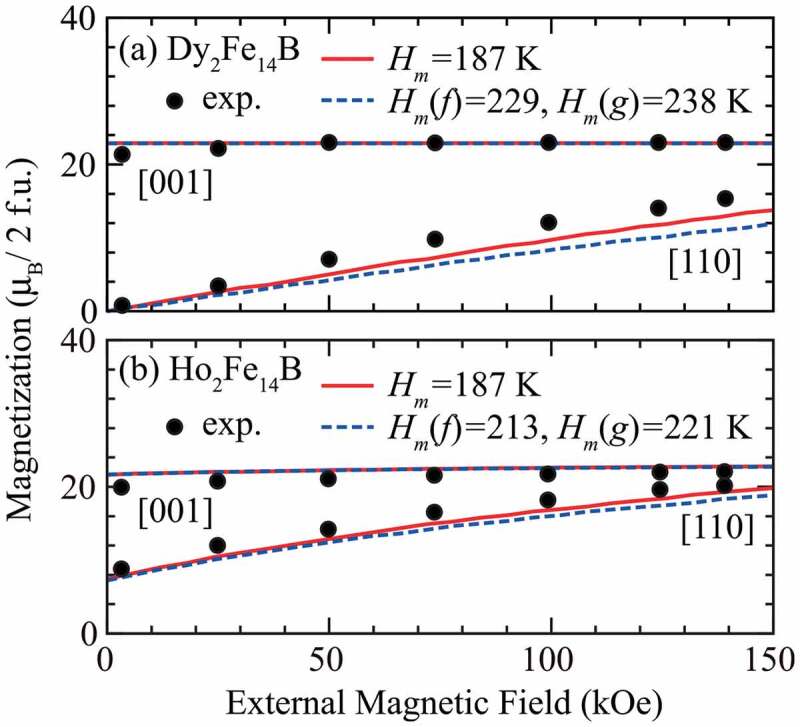
Magnetization curves of $R_{2}$Fe${\;}_{14}$B (R=Dy, Ho). Solid and broken lines represent the results calculated using the molecular field $H_{m}$ determined from the experimental results [22] and the first-principles calculations, respectively. Filled circles represent experimental results reproduced from [12]. (Reprinted with permission from [3]. Copyright 2018 by the American Physical Society.)

Recently, the first two authors of this review confirmed that the effective spin model description performs well for heavier rare-earth intermetallic compounds Dy${\;}_{2}$Fe${\;}_{14}$B and Ho${\;}_{2}$Fe${\;}_{14}$B [3]. Figure 1 shows the magnetization curves of these materials along the [001] and [110] axes at T=4.2 K. The solid and broken lines depict theoretical results obtained using the effective spin model, whereas the filled circles depict experimental data reproduced from [12]. We observe that the theoretical and experimental results are in very good agreement, which suggests the validity of the effective spin model for these compounds. Notably, the magnetization along the [110] direction is zero for Dy${\;}_{2}$Fe${\;}_{14}$B in the absence of an external magnetic field but non-zero for Ho${\;}_{2}$Fe${\;}_{14}$B. This is a manifestation of the so-called spin-reorientation phenomenon, which is only observed in Ho${\;}_{2}$Fe${\;}_{14}$B for these two materials. We further confirmed that the temperature dependence of the tilt angle of the magnetization under the zero-field in Ho${\;}_{2}$Fe${\;}_{14}$B is also well described by the effective spin model [3]. Thus, we may conclude that the effective spin model successfully describes the finite temperature magnetic properties of Dy${\;}_{2}$Fe${\;}_{14}$B and Ho${\;}_{2}$Fe${\;}_{14}$B, and reflects the differences in rare-earth elements.

**Figure 2.**
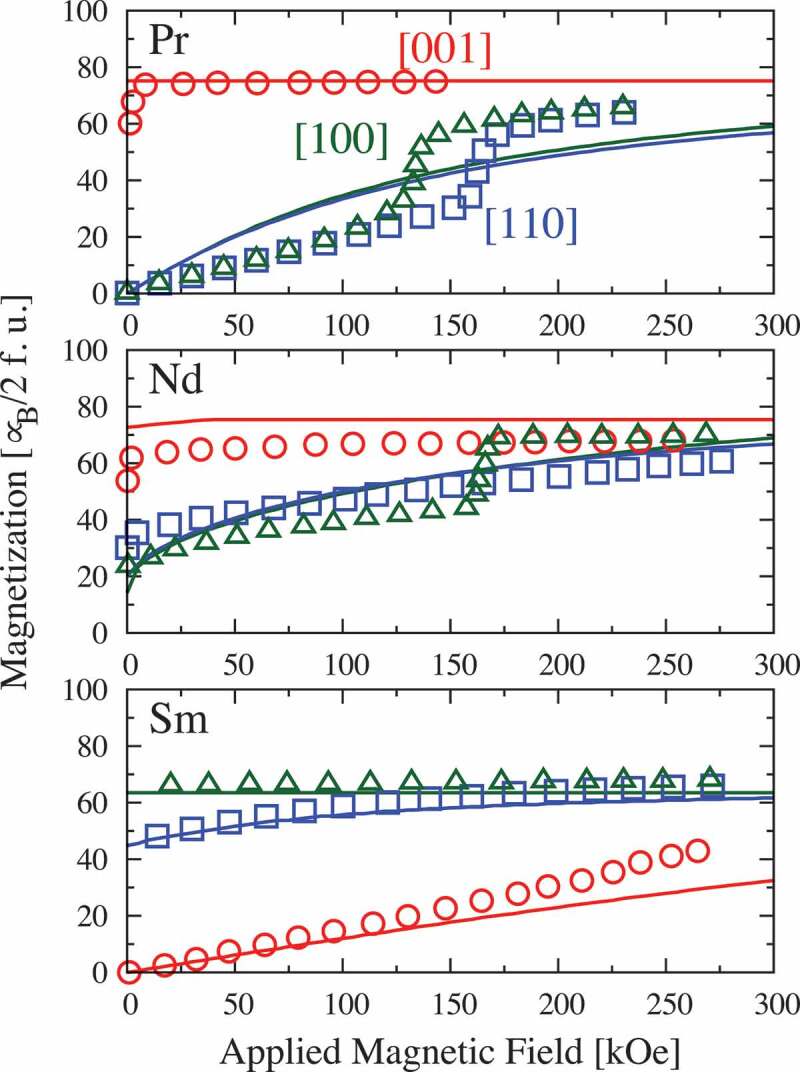
Magnetization curves of $R_{2}$Fe${\;}_{14}$B (R=Pr, Nd, Sm) along the [001] (red), [100] (green), and [110] (blue) axes. Open circles represent experimental results [12]

Next, we consider the results for $R_{2}$Fe${\;}_{14}$B with lighter rare-earth elements, such as R=Pr, Nd, and Sm. Figure 2 shows the magnetization curves of these materials along the [001], [100], and [110] directions at T=4.2 K. Here, the solid lines represent theoretical results, and the open symbols represent the experimental results reproduced from [12]. The effective spin model is in good agreement with the experimental results only for Sm${\;}_{2}$Fe${\;}_{14}$B; for Pr${\;}_{2}$Fe${\;}_{14}$B and Nd${\;}_{2}$Fe${\;}_{14}$B, discrepancies with the experimental results are noted at a qualitative level, such as its failure to describe first-order magnetization processes (FOMP) along the [100] and [110] directions. This must be considered when applying the effective spin model to lighter rare-earth intermetallic compounds. However, we note that the behavior that cannot be described by the effective spin model, such as the FOMP, is observed only at low temperatures, and we have also confirmed that the effective spin model description is practically sufficient above room temperature.

**Figure 3.**
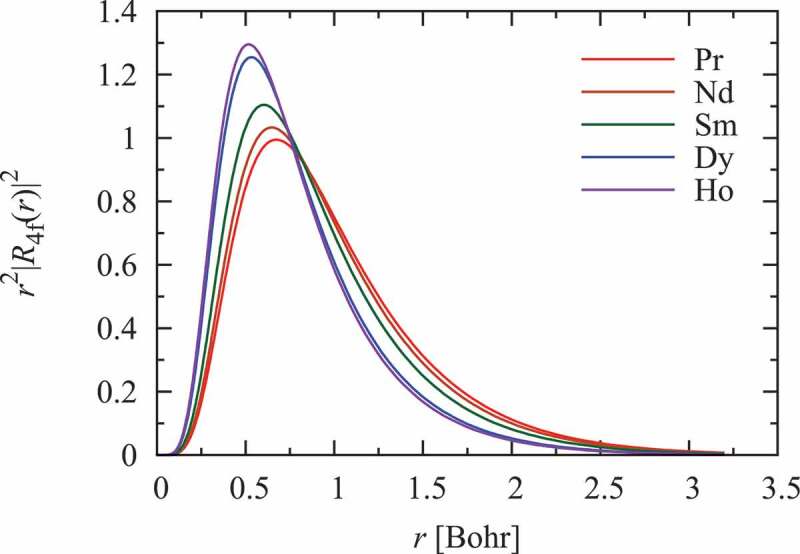
Radial 4*f* charge densities $r^{2}{\left|R_{4f}\right|}^{2}$ of Pr, Nd, Sm, Dy, and Ho, which were obtained by performing atomic calculations of the electron structure of isolated rare-earth atoms. In these calculations, the self-interaction correction (SIC) was included, which leads to a better approximation for the single electron densities. This approach provided a 4*f* charge density that was very close to that obtained using more rigorous SIC-DFT band calculations [1]

The spin model does not precisely describe the magnetic properties of lighter rare-earth intermetallic compounds at low temperatures, especially those of Pr and Nd, because 4*f* electrons of these ions are less tightly bound to the nucleus. This situation is clearly presented in Figure 3, which shows the radial shapes of the 4*f* charge densities of Pr, Nd, Sm, Dy, and Ho, which were obtained through atomic calculations of the electron structures of isolated rare-earth atoms. In the case of these very light rare-earth elements, it is evident that the preconditions for applying the open-core method, which is the basis for constructing an effective spin model, are not completely satisfied. Therefore, a novel method to describe weakly localized 4*f* electrons is required to construct a CF Hamiltonian. A promising method was recently developed by one of the authors of this review (P. N). It was first applied to rare-earth aluminates or oxides, and it yielded excellent results [23]. Making this method applicable to magnetic rare-earth intermetallic compounds is not a straightforward task; however, several attempts have been, and are still being, made [24].

### 3.2. SmFe_12_

There are three types of Sm compounds that are used as practical permanent magnetic materials, namely SmCo${\;}_{5}$, Sm${\;}_{2}$Co${\;}_{17}$, and Sm${\;}_{2}$Fe${\;}_{17}$N${\;}_{3}$. Recently, SmFe${\;}_{12}$ has attracted renewed interest as a candidate for a new rare-earth lean permanent magnetic material because it exhibits excellent intrinsic magnetic properties, such as uniaxial magnetic anisotropy. Thus, it is important to establish an effective spin model method for Sm-based intermetallic compounds for the future development of new permanent magnets.

**Figure 4.**
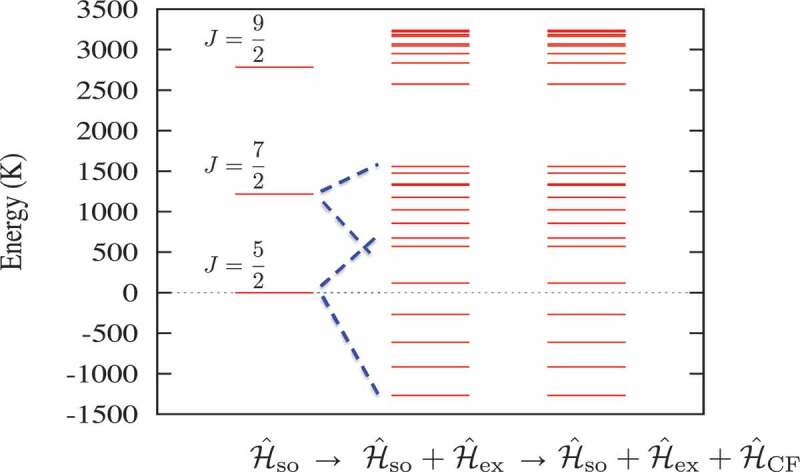
Calculated energy levels of the Sm 4*f* electron states in SmFe${\;}_{12}$ in the absence of an external magnetic field. The energy levels determined by the spin-orbit Hamiltonian $H_{so}$ are split by the strong exchange field $H_{ex}$, which acts on the spins of the 4*f* electrons, thereby resulting in the level crossing of states with different quantum numbers J. However, the CF Hamiltonian $H_{CF}$ only slightly shifts these energy levels

Here, we return to the Hamiltonian for the effective spin model given in Equations (1) and (2). For free trivalent rare-earth ions, the low-lying electron states are well described by the Russell–Saunders coupling scheme. The orbital angular momentum L and spin S in the ground state are given by Hund’s rule; the total angular momentum J is J=L−S for light rare-earth elements. Because the spin-orbit coupling of the lanthanide 4*f* orbitals is strong (3000–15,000 K), only the ground state of the J-multiplet needs to be considered during the calculation. For such a case, we may use the truncated Hamiltonian (2). However, Eu${\;}^{3+}$ and Sm${\;}^{3+}$ are well-known exceptions, where the first excited state of the J-multiplet is at 366 and 1340 K above the ground state, respectively. Furthermore, when the ions are in a permanent magnet, the strong exchange field acting on the 4*f* electron spins leads to a mixture of states with different quantum numbers J, as shown in Figure 4. Therefore, it is no longer possible to restrict the Hilbert space of the Hamiltonian to a subspace with the lowest J-multiplet, and the Hamiltonian in Equation (1) must be used for the effective spin model of Sm-based compounds.

**Figure 5.**
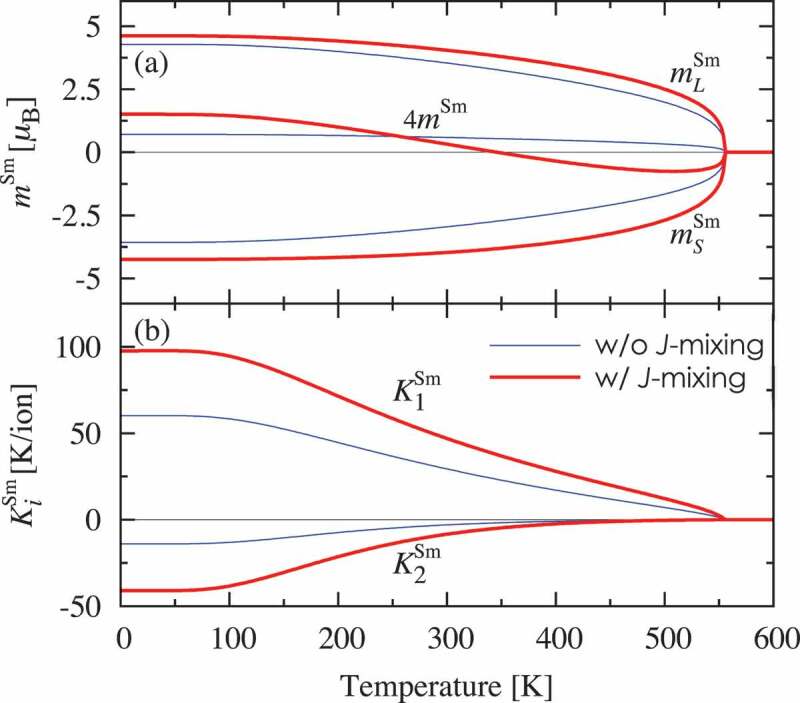
Temperature dependences of the (a) orbital ($m_{L}^{Sm}$), spin ($m_{S}^{Sm}$), and total ($m^{Sm}$) magnetic moments in the absence of an external magnetic field and (b) magnetic anisotropy constants per Sm ion $K_{1,2}^{Sm}$ in SmFe${\;}_{12}$, which were obtained using the effective spin model

Next, let us examine the differences between the two Hamiltonians (1) and (2) in the description of the magnetic properties; we use SmFe${\;}_{12}$ as an example. Figure 5 shows the temperature dependences of the orbital, spin, and total magnetic moments of the Sm ion and the magnetic anisotropy constant $K_{1}$ and $K_{2}$ for each Sm ion, which were calculated using the effective spin models with a truncated Hamiltonian (2) or an unrestricted one (1). The results show that the J-mixing effects increase the absolute values of both $m_{L,S}$ and $K_{1,2}$ over the entire temperature range. We observe that the total magnetic moment of the Sm ion m is reversed at approximately T=350 K. Notably, the effective spin model with an unrestricted Hamiltonian (1) provides good descriptions for the finite temperature magnetic properties of SmFe${\;}_{12}$ and Sm${\;}_{2}$Fe${\;}_{17}$N${\;}_{3}$, as reported in [25] and [26], respectively.

## 4. Surface magnetic properties

In this section, we discuss the surface magnetic anisotropy of the crystalline Nd${\;}_{2}$Fe${\;}_{14}$B and SmFe${\;}_{12}$, and we estimate the switching field of the fine particles of these materials. First, we consider the CF parameters $A_{l}^{m}\langle r^{l}\rangle$ of rare-earth ions in Nd${\;}_{2}$Fe${\;}_{14}$B and SmFe${\;}_{12}$, which are calculated using (3) on the basis of first-principles calculations in the following two subsections. To obtain $V_{l}^{m}(r)$ and $R_{4f}(r)$ in (3), we use the full-potential linearized augmented plane-wave plus local orbital method (APW+lo), which was implemented using WIEN2k code [27]. Furthermore, we carry out separate atomic calculations of the electron structure of isolated Nd and Sm atoms using the same approach as that in our previous study. Here, the lattice constants of the primitive cell are set to the experimental values of a=b=8.80Å and c=12.19Å for Nd${\;}_{2}$Fe${\;}_{14}$B [28], and a=b=8.35Å and c=4.8Å for SmFe${\;}_{12}$ [29]. Throughout this study, we use $R_{MT}=3.2$, 2.09, and 1.85 a.u. for the Nd, Fe, and B ions in Nd${\;}_{2}$Fe${\;}_{14}$B, respectively, and $R_{MT}=3.2$ and 2.21 a.u. for the Sm and Fe ions in SmFe${\;}_{12}$, respectively. The computational details are provided in Ref [2].

### Nd${\;}_{2}$Fe${\;}_{14}$B

4.1.

In Table 1, we summarize the CF parameter $A_{2}^{0}\langle r^{2}\rangle$ of the Nd ions in the bulk system at the (100) and (001) surfaces of the Nd${\;}_{2}$Fe${\;}_{14}$B structure [2,8]. We observe that $A_{2}^{0}\langle r^{2}\rangle$ is negative only when the Nd ion is on the (001) surface. Because $A_{2}^{0}\langle r^{2}\rangle$ is the leading term of the CF parameters, its sign is the main factor that determines the magnetic anisotropy of rare-earth ions. A positive $A_{2}^{0}\langle r^{2}\rangle$ indicates that the Nd ions exhibit uniaxial magnetic anisotropy. Therefore, we observe that Nd ions on the (001) surface exhibit planar magnetic anisotropy.

**Table 1.** CF parameter $A_{2}^{0}\langle r^{2}\rangle$ [K] of Nd ions in the crystalline N$d_{2}$F$e_{14}$B structure

Nd site	Bulk	(100)	(001)
f	463	640, 479	−413
g	358	432, 590	−432

**Figure 6.**
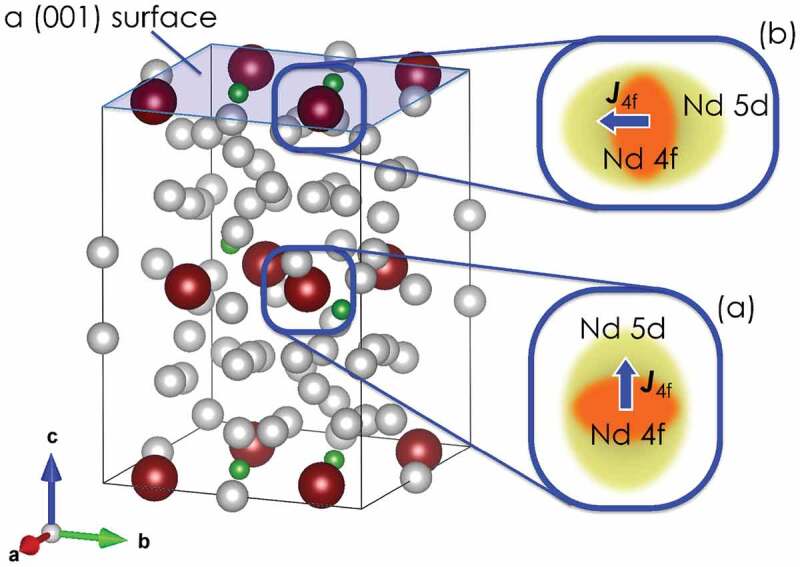
Nd${\;}_{2}$Fe${\;}_{14}$B structure with a (001) surface

We now discuss the physical reason for the planar magnetic anisotropy of Nd ions on the (001) surface. Intuitively, the uniaxial magnetic anisotropy of Nd${\;}_{2}$Fe${\;}_{14}$B along the c-axis can be explained as follows. In the Nd${\;}_{2}$Fe${\;}_{14}$B structure, the nearest-neighboring ions of each Nd ion are the Fe ions located in the direction slightly tilted from the c-axis, as observed from the Nd ion. Thus, 5d or 6p valence electrons of the Nd ions are primarily exchange-coupled with 3*d* electrons of this Fe, which results in a slightly extended distribution of valence electrons along the c-axis. Then, the 4*f* electron cloud of the Nd ion tends to avoid any overlap with the prolate valence electron clouds to reduce the electrostatic energy between them; it also has a slightly flat distribution along the c-axis, as shown in the inset (a) of Figure 6. The magnetic moment $J_{4f}$ of the 4*f* electrons is also depicted by a thick blue arrow in the same inset. Thus, this magnetic moment is energetically favorable when it is directed toward the c-axis. This is an intuitive explanation of the c-axis magnetic anisotropy of Nd ions in the Nd${\;}_{2}$Fe${\;}_{14}$B structure.

This scenario also holds when Nd ions are on the (100) surface, because neighboring Fe ions exist for each Nd ion on these surfaces. However, if an Nd ion is exposed to the (001) surface, it loses the nearest-neighboring Fe ions. Thus, the valence charge distribution of the Nd ion shifts from prolate to oblate to hybridize with the orbitals of the next-nearest ions, which results in planar magnetic anisotropy, as shown in the magnified image of inset (b) in Figure 6.

### 4.2. SmFe_12_

Next, we investigate the CF parameters $A_{l}^{m}\langle r^{l}\rangle$ of Sm ions in the bulk system on both the (100) and (001) surfaces of SmFe${\;}_{12}$. We list $A_{l}^{m}\langle r^{l}\rangle$ up to the fourth order in Table 2. Here, we observe that the leading term $A_{2}^{0}\langle r^{2}\rangle$ is negative when Sm ions are in the bulk system and at the (001) surface. Because the sign of the second-order Stevens factor of Sm${\;}^{3+}$ is positive, the negative $A_{2}^{0}\langle r^{2}\rangle$ value indicates that Sm ions exhibit uniaxial magnetic anisotropy along the c-axis. For the (100) surface, we note that $A_{2}^{0}\langle r^{2}\rangle$ is positive and that some additional terms of $A_{l}^{m}\langle r^{l}\rangle$ appear (that is, $A_{2}^{2}\langle r^{2}\rangle$ and $A_{4}^{2}\langle r^{4}\rangle$) owing to the breaking of the original I4/mmm symmetry. Interestingly, $A_{2}^{2}\langle r^{2}\rangle$ has a fairly large negative value. We note that this term affects the anisotropy parameter ${\tilde{K}}_{1}(\phi ,T)$ in (9) via $K{{\;}^{\prime }}_{1}(T)$ in (13), and it contributes to the planar magnetic anisotropy of Sm ions on the (100) surface.

**Table 2.** CF parameter $A_{l}^{m}\langle r^{l}\rangle$ [K] of the Sm ions in SmFe${\;}_{12}$, which were obtained using first-principles calculations

Sm site	$A_{2}^{0}\langle r^{2}\rangle$	$A_{2}^{2}\langle r^{2}\rangle$	$A_{4}^{0}\langle r^{4}\rangle$	$A_{4}^{2}\langle r^{4}\rangle$	$A_{4}^{4}\langle r^{4}\rangle$
bulk	−71.4	–	−21.3	–	−49.3
(100)	47.2	−1304.2	−15.8	−106.0	70.8
(001)	−895.6	–	2.44	–	68.1

**Figure 7.**
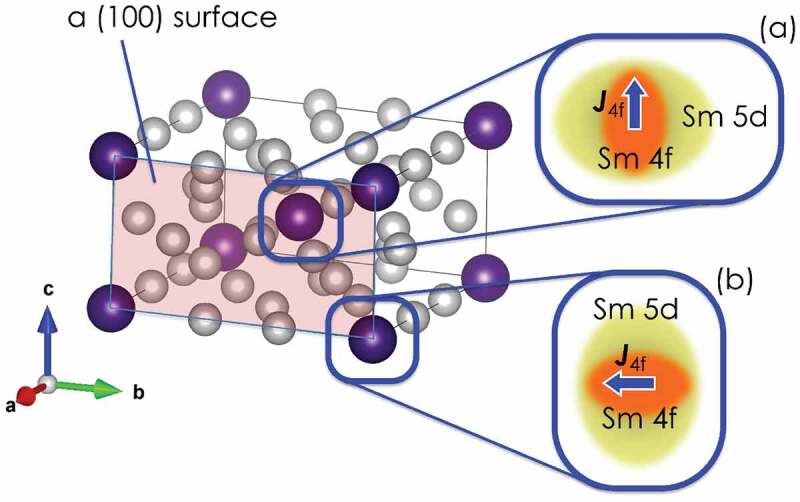
SmFe${\;}_{12}$ with a (100) surface

We provide an intuitive explanation for the planar magnetic anisotropy of Sm ions on the (100) surface, as in the Nd${\;}_{2}$Fe${\;}_{14}$B case. In the ThMn${\;}_{12}$ structure, the nearest-neighboring ions of each Sm ion are the Fe ions on the so-called 8i-site in the same c-plane. Thus, the valence electron cloud of Sm ions has an oblate distribution, and the 4*f* electron cloud with a positive Stevens factor tends to extend along the c-axis to avoid overlapping with the valence electron cloud, as shown in the inset (a) of Figure 7. This situation is reversed if Sm ions are on the (100) surface.

### 4.3. Surface magnetic anisotropy and switching fields

We discuss the surface magnetic anisotropy owing to the planar local anisotropy, which is caused by surface Nd or Sm ions. We compute the magnetic anisotropy energy of a unit cell including two formula units (f.u.) of Nd${\;}_{2}$Fe${\;}_{14}$B or SmFe${\;}_{12}$ based on Equation (8) by using the information presented in the previous two subsections.

**Figure 8.**
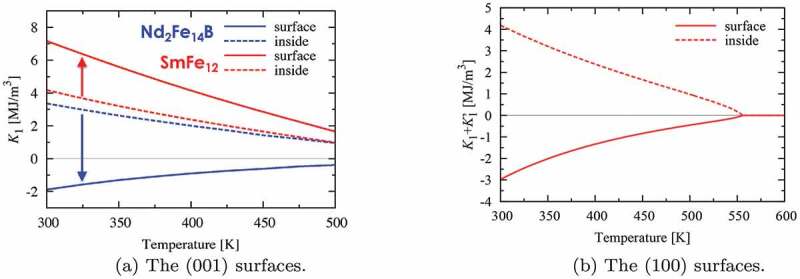
Magnetic anisotropy constants of each unit cell including two f.u. of Nd${\;}_{2}$Fe${\;}_{14}$B (blue) or SmFe${\;}_{12}$ (red) as a function of temperature. $K_{1}$ is negative for (a) the unit cell of Nd${\;}_{2}$Fe${\;}_{14}$B on the (001) surface and for (b) $K_{1}+K{{\;}^{\prime }}_{1}$ of SmFe${\;}_{12}$ on the (100) surface

Figure 8 shows the magnetic anisotropy constant $K_{1}$ of each unit cell on (a) the (001) surfaces and (b) the (100) surfaces, as well as inside the materials, as a function of temperature. In Figure 8(a), we clearly observe that the $K_{1}$ value of the Nd${\;}_{2}$Fe${\;}_{14}$B unit cell on the (001) surface decreases significantly and becomes negative at all temperatures. By contrast, the $K_{1}$ value of the SmFe${\;}_{12}$ unit cell increases when the unit cell is on the (001) surface; however, it decreases on the (100) surface, which is expected from the $A_{2}^{0}\langle r^{2}\rangle$ of the Sm ions on these surfaces, as shown in Table 2.

**Figure 9.**
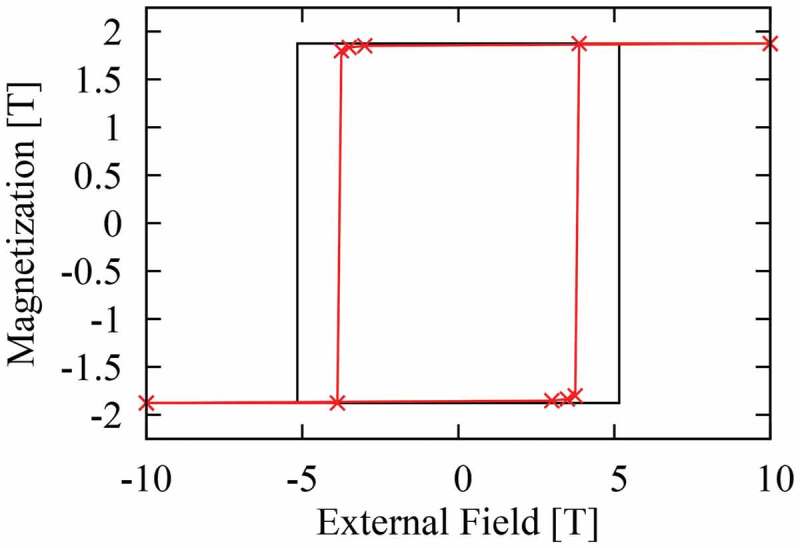
The magnetization curves of a SmFe${\;}_{12}$ fine particle obtained using the atomistic spin model at T=300 K. It can be observed that the switching field is reduced to approximately 70% of the anisotropy field when the surface effect is considered (red line)

The surface magnetic anisotropy with negative $K_{1}$, as described above, may be partly responsible for a reduction of the switching field of a Nd${\;}_{2}$Fe${\;}_{14}$B or SmFe${\;}_{12}$ fine particle. In a previous study, we showed that for Nd${\;}_{2}$Fe${\;}_{14}$B, such surface magnetic anisotropy does indeed reduce the switching field by approximately 30% [2]. Here, we investigate the same effects for SmFe${\;}_{12}$. First, we use the atomistic spin model, which was introduced in Section 2, to estimate the switching field of a small cluster of SmFe${\;}_{12}$ by solving Equation (7). The magnetization curve obtained at T=300 K is shown in Figure 9. The black line represents the magnetization curve without the surface effects and the demagnetization effects. If we consider the planar magnetic anisotropy of the Sm ions on the (100) surface, we obtain the magnetization curve shown by the red line. This figure shows that the reduction of the SmFe${\;}_{12}$ switching field by surface effects is approximately 30%.

**Figure 10.**
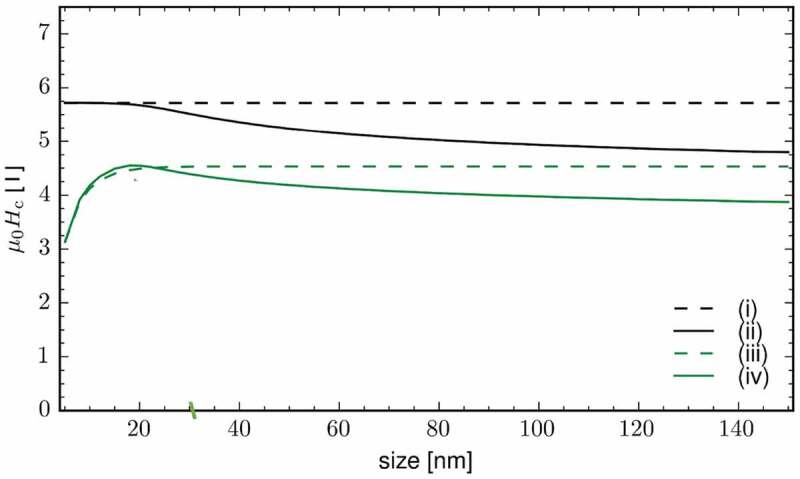
Size dependences of the switching field of a cubic model (i) without both the surface and the demagnetizing field effects, (ii) with only the demagnetizing field effect, (iii) with only the surface effect, and (iv) with both effects. We observe that the switching field is reduced by 20% due to the surface effect alone, even if the system size is larger than 100 nm

However, it is intuitive to suspect that the surface effects may disappear as the system size increases. Therefore, we study the size dependence of the switching field reduction, owing to the surface magnetic anisotropy effects. To this end, the atomistic spin model is not useful because it has excessive spin degrees of freedom, and it requires significant computational resources to simulate the magnetization reversal processes of a fine particle larger than a few tens of nanometers. Therefore, a coarse-grained model is required for this simulation. Establishing a general method to develop a coarse-grained atomistic spin model is a major challenge. Here, we make a simple and approximate attempt to obtain a coarse-grained micromagnetic model. A single SmFe${\;}_{12}$ unit cell is approximately the same size as the numerical unit cell in modern micromagnetic simulation models; thus, we use the anisotropy constant $K_{1}$ shown in Figure 8 as a model parameter in the present micromagnetic simulation. For micromagnetic energy minimization [30], the total Gibbs free energy is augmented with a surface energy term. Here, we only show the results of the size dependence of the switching field of a cubic micromagnetic model obtained via the present coarse-grain treatment. Figure 10 shows the switching field, or coercivity, of the cubic model as a function of the linear size of the system. We can clearly observe that the switching field is reduced by 20% only due to the surface effect, even if the system size is larger than 100 nm.

## 5. Summary

We present a review of rare-earth permanent magnetic materials, focusing on the theoretical aspects based on first-principles calculations and statistical mechanical analyses using the effective spin models. Equilibrium magnetic properties, such as the magnetization curves of $R_{2}$Fe${\;}_{14}$B (R = Pr, Nd, Sm, Dy, Ho) and SmFe${\;}_{12}$, are well described by the effective spin model approach. Furthermore, we studied a possible reduction mechanism in the switching field of Nd${\;}_{2}$Fe${\;}_{14}$B or SmFe${\;}_{12}$ fine particles, owing to the surface magnetic anisotropy, by using the atomistic spin model based on first-principles calculations and the continuum micromagnetic model. We confirmed that the switching field of these systems is reduced by 20% of the anisotropy field $H_{A}$, even when the system size is larger than 100 nm.
